# Efficacy and patient-reported outcomes in advanced non-small cell lung cancer patients receiving aumolertinib as first-line therapy: a real-world study

**DOI:** 10.3389/fphar.2024.1444707

**Published:** 2024-09-06

**Authors:** Hongxin Li, Wen Zhao, Caiyun Chang, Tiantian Xuan, Chengjun Wang, Rongyu Zhang, Chuang Yang, Jian Wang, Cuihua Yi, Xiuwen Wang, Shuwen Yu, Jisheng Li

**Affiliations:** ^1^ School of Pharmaceutical Sciences, Cheeloo College of Medicine, Shandong University, Jinan, Shandong, China; ^2^ Department of Medical Oncology, Qilu Hospital, Cheeloo College of Medicine, Shandong University, Jinan, Shandong, China; ^3^ Department for Infectious Disease Control and Prevention, Jinan Municipal Center for Disease Control and Prevention, Jinan, Shandong, China; ^4^ Department of Medical Oncology, Qilu Hospital (Qingdao), Cheeloo College of Medicine, Shandong University, Qingdao, Shandong, China; ^5^ Department of Pharmacy, Qilu Hospital, Cheeloo College of Medicine, Shandong University, Jinan, Shandong, China; ^6^ Clinical Trial Center, NMPA Key Laboratory for Clinical Research and Evaluation of Innovative Drugs, Shandong University, Jinan, Shandong, China

**Keywords:** non-small cell lung cancer, epidermal growth factor receptor, aumolertinib, patient-reported outcomes, efficacy

## Abstract

**Background:**

Aumolertinib demonstrated superior progression-free survival (PFS) and a well-tolerated toxicity profile compared to gefitinib in front-line treatment of locally advanced or metastatic non-small cell lung cancer (NSCLC) in the AENEAS trial. However, patient-reported outcomes (PROs) of aumolertinib have not been published.

**Methods:**

In this real-world study, the efficacy was evaluated by Response Evaluation Criteria in Solid Tumors (RECIST) 1.0. PROs were evaluated using the European Organization for Research and Treatment of Cancer Quality of Life Questionnaire C30 (QLQ-C30) and the EORTC Quality of Life lung cancer-specific module (QLQ-LC13) in advanced NSCLC patients receiving aumolertinib as initial therapy. Pre-specified key symptoms were cough, hemoptysis, dyspnea, sore mouth or tongue, dysphagia, hair loss, tingling in hands or feet, chest pain, arm or shoulder pain, and pain at other sites.

**Results:**

A total of 33 patients were included, 23 of whom had efficacy information up to January 2024. The median follow-up time was 264 days (interval: 36–491 days). The objective response rate and disease control rate were 65.2% and 91.3%, respectively. The EORTC QLQ-LC30 general health status scale showed that functional scales increased and symptom scales decreased during aumolertinib treatment. Symptom scales assessed by the EORTC QLQ-LC13 showed that improvements in cough, sore mouth or tongue, tingling in hands or feet, chest pain, arm or shoulder pain, and other pain sites were both clinically and statistically significant after 6 months of aumolertinib treatment (*p* < 0.05).

**Conclusion:**

In this real-world study, aumolertinib showed comparable disease control and objective response rates as reported in the AENEAS trial for advanced NSCLC patients with EGFR-sensitizing mutations. Aumolertinib treatment improved PROs, further supporting it in first-line clinical practice.

## 1 Introduction

Worldwide, lung cancer ranks first in cancer-related deaths, of which non-small cell lung cancer (NSCLC) accounts for approximately 85% (Travis et al., 2015). The discovery of an epidermal growth factor receptor (EGFR)-sensitive mutation and the development of epidermal growth factor receptor tyrosine kinase inhibitors (EGFR-TKIs) pioneered targeted therapy for NSCLC. EGFR-TKIs, including the first, second, and third-generation drugs, significantly prolonged progression-free survival (PFS) and overall survival (OS) for advanced NSCLC patients with sensitive EGFR mutations as first-line treatment (Zhou et al., 2011; Maemondo et al., 2010; Fukuoka et al., 2011; Wu et al., 2014; Yang et al., 2015; Wu et al., 2017; Mok et al., 2017).

Advanced NSCLC is characterized by a high symptom burden (Iyer et al., 2014). At least 90% of patients experience fatigue, appetite loss, dyspnea, and pain, which significantly negatively impact disease-specific health-related quality of life (HRQoL) (Iyer et al., 2013; Polanski et al., 2016). Knowledge of the effects of new therapies on patient experiences, when combined with survival data, can provide crucial information to assist physicians and patients in making informed treatment decisions (Bottomley et al., 2005; Fallowfield and Fleissig, 2011). Compared with chemotherapy, the first-generation EGFR-TKIs, including gefitinib and erlotinib, significantly improved symptom control and HRQoL. Afatinib, a representative of the second generation EGFR-TKIs, exhibited similar results (Chen et al., 2013; Geater et al., 2015; Oizumi et al., 2012). In the ARCHER 1050 trial, dacomitinib, when used as first-line treatment for NSCLC, demonstrated superior survival compared to gefitinib. However, global HRQoL improvements were observed only with gefitinib (Wu et al., 2017). Osimertinib, a third-generation EGFR-TKI, demonstrated superior survival outcomes compared to first-generation EGFR-TKIs (Soria et al., 2018). Investigators sought to determine whether osimertinib provided better patient-reported outcomes (PROs) in addition to its longer survival benefits. However, PRO results from the FLAURA trial revealed that key symptoms improved significantly and were clinically relevant in both the osimertinib and erlotinib/gefitinib arms (Leighl et al., 2020). These findings suggest that the efficacy data reported by investigators may not fully align with PROs. It is, therefore, recommended that PROs and HRQoL be assessed in all prospective clinical comparative effectiveness research studies (Bottomley et al., 2005).

Aumolertinib (HS-10296) is a novel, irreversible, third-generation EGFR-TKI targeting both EGFR-sensitizing and T790M mutations while sparing wild-type EGFR. In the APOLLO registrational trial, patients with EGFR T790M-positive advanced NSCLC after disease progression on a first- or second-generation EGFR-TKI achieved a median PFS of 12.4 months, and the toxicity profile was tolerable (Lu et al., 2022a). ANEAS, a randomized, double-blind, phase-III trial, evaluated the efficacy and safety of aumolertinib compared with gefitinib as a first-line treatment of locally advanced or metastatic EGFR-mutated NSCLC. Aumolertinib achieved better survival than gefitinib, with a median PFS of 19.3 months *versus* 9.9 months (hazard ratio, 0.46; 95% CI, 0.36 to 0.60; *p* < 0.0001) (Lu et al., 2022b). Based on these results, aumolertinib was approved in China to treat advanced NSCLC with EGFR-sensitizing and T790M mutations.

Several studies also demonstrated the efficacy and safety profile of aumolertinib in real-world settings (Zhang et al., 2024; Ding et al., 2022; Zhang et al., 2022). However, all of these studies were retrospective, and some only reported individual cases. Importantly, PRO changes during aumolertinib treatment have not been reported. We prospectively collected European Organization for Research and Treatment of Cancer (EORTC) Quality of Life Questionnaire C30 (QLQ-C30) and EORTC Quality of Life lung cancer-specific module (QLQ-LC13) information and efficacy data. This showed that aumolertinib treatment significantly improved HRQoL.

## 2 Methods

### 2.1 Patients and study design

This prospective study was conducted between September 2022 and January 2024. Eligible patients were aged 18 years or older, with histologically/cytologically confirmed locally advanced or metastatic NSCLC, carrying an EGFR mutation, and having not received previous systemic anticancer therapy. The exclusion criteria were (i) previous receipt of any systemic therapy; (ii) concurrent presence of other malignancies requiring active treatment; and (iii) any other condition that, in the investigator’s judgment, rendered the patient unsuitable for participation in this study. Enrolled patients received oral aumolertinib 110 mg once daily until disease progression, intolerable toxicity, or a request to discontinue by the patient or physician.

The treatment response was evaluated according to the Response Evaluation Criteria in Solid Tumors (RECIST) version 1.0 based on computed tomography (CT) imaging. HRQoL was assessed with the use of the self-administered cancer-specific European Organization for Research and Treatment of Cancer (EORTC) quality-of-life questionnaire C30 (QLQ-C30) and its lung cancer-specific module, the QLQ-LC13. Patients were assessed monthly for the first half of the year from the start of treatment and then every 3 months until the 18th month. The primary endpoint was HRQoL. Secondary endpoints included objective tumor response (ORR) and disease control rate (DCR).

This study complied with the Ethical Guidelines for Medical and Health Research Involving Human Subjects. The study protocol received approval from the Ethical Review Boards and Institutional Review Boards of Qilu Hospital of Shandong University (KYLL-202308-041). All patients provided written, informed consent.

### 2.2 Assessment of tumor response and effectiveness

Tumor response was determined according to RECIST1.0 and was assessed every 2 months until disease progression. The objective response rate (ORR) was defined as the percentage of patients with a tumor-confirmed overall response of complete response (CR) or partial response (PR) in the total number of patients analyzed. The disease control rate (DCR) was defined as the percentage of patients with a tumor-confirmed overall response of complete CR, PR, or stable disease (SD). Progression-free survival (PFS) was followed up until the date of the first tumor progression or death for any reason, whichever occurred first. Overall survival (OS) was followed up until death for any reason.

### 2.3 EORTC QLQ-C30 and EORTC QLQ-LC13

The EORTC QLQ-C30 included five functional scales (physical, role, cognitive, emotional, and social), three symptom scales (fatigue, pain, nausea, and vomiting), and the general health status scale. Multiple individual items on other common symptoms of cancer (dyspnea, loss of appetite, insomnia, constipation, and diarrhea) were also assessed, as were individual items measuring the economic impact of the disease. The majority of items were reported on verbal response scales of 1–4 with response options of “not at all,” “a little bit,” “quite a bit”, and “very much,” while the two general health status items were reported on numeric response scales of 1–7 with endings of “very poor” and “excellent”.

The EORTC QLQ-LC13 consists of 13 questions on a multi-item scale, including questions measuring lung-cancer-related symptoms (coughing, hemoptysis, and dyspnea) and treatment-related adverse effects (sore mouth or tongue, dysphagia, hair loss, tingling in the hands or feet, chest pain, arm or shoulder pain, other pain, and the usefulness of pain medication). The QLQ-LC 13 item uses the same 1–4 verbal response scale as the QLQ-C30 item.

For each scale or item, a linear transformation was applied to normalize the raw score to 0–100. Higher scores on the functional and general health status scales represented better health status, while the opposite was true for the symptom scales. Any score change of 10 points from baseline was considered to be clinically meaningful. Improvement in health status was defined as an increase of ≥10 points from baseline in functional scale scores and a decrease of ≥10 points in symptom scales/items. Deterioration was defined as a decrease of ≥10 points in functional scales and an increase of ≥10 points in symptom scales/items. Otherwise, they were considered stable.

### 2.4 Statistical analyses

Descriptive statistical analysis of demographic information and clinical characteristics was performed, and chi-square testing was used to verify whether the distribution of the parameters conformed to a normal distribution. Differences between groups were assessed by ANOVA and Kruskal–Wallis’s test. The Kaplan–Meier method was used to estimate the median PFS and OS with 95% confidence intervals (CIs). The questionnaire scales/items were scored according to the EORTC-published algorithm. Mean QLQ-C30 or QLQ-LC 13 scales or individual item scores and criteria were calculated at all time points to characterize patient efficacy after amitriptyline treatment (a 10-point difference between the score at each time point; the first month’s score was considered clinically significant). Statistical analysis and plotting were performed using SPSS version 27 (IBM, Chicago, IL, United States) and GraphPad Prism version 9.5.1 (San Diego, California, United States).

## 3 Results

### 3.1 Patient characteristics

**FIGURE 1:**
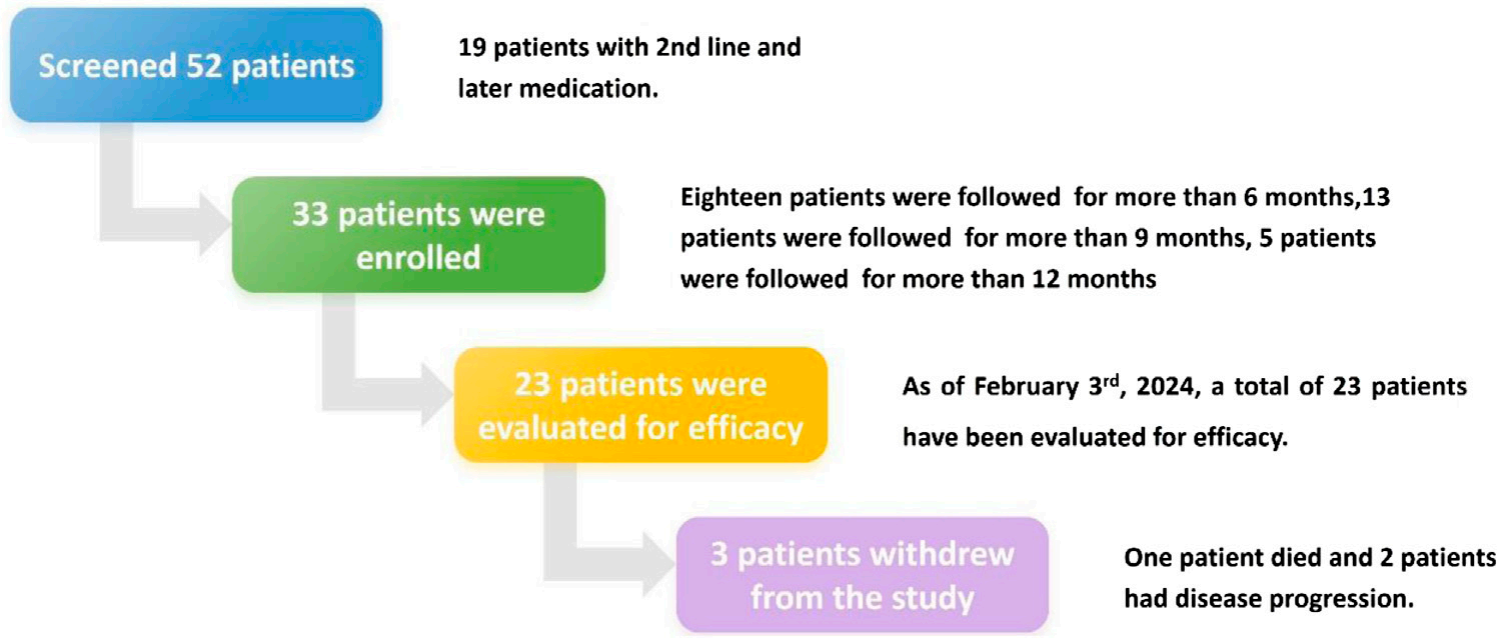
Flow chart of the research process.

A total of 52 patients diagnosed with EGFR-mutated (Exon 19 deletion or Exon 21 L858R) locally advanced or metastatic NSCLC and receiving aumolertinib treatment were successively screened from September 2022 (Figure 1). Of the 52 patients screened, 19 received aumolertinib as second- or later-line treatment; finally, 33 patients were enrolled. During the course of the study, three cases dropped out. Of these, one patient passed away after 10 months of medication (it is unclear whether the death was related to the illness), and two withdrew from the study due to disease progression and switched to alternative treatments.

Patient demographics and clinicopathological characteristics are presented in Table 1. The median age at diagnosis was 63 years (range: 38–83). The cohort included 21 female patients (63.6%) and 12 males (36.4%). Among the patients, six (18.2%) were former smokers, while 27 (81.8%) had never smoked. The majority of patients (81.8%) had an ECOG-PS of 0 or 1. The proportions of EGFR L858R and EGFR 19DEL mutations were 42.4% and 39.4%, respectively. Liver metastasis, brain metastasis, and bone metastasis were observed in 15.2%, 30.3%, and 51.5% of patients, respectively.

**TABLE 1:** Patient demographics and baseline characteristics.

Characteristics	N (%)
Patients	33 (100)
Men	12 (36.4)
Women	21 (63.6)
Age (years), median (range)	63 (38–83)
Stage	
IV	33 (100)
Smoking history	
Ever Never	6 (18.2) 27 (81.8)
ECOG PS	
0–1 2 Unknown	27 (81.8) 5 (15.2) 1 (3.0)
Genetic mutation	
EGFR L858R EGFR 19Del EGFR G719X Unknown	13 (39.4) 14 (42.4) 1 (3.0) 5 (15.2)
Metastasis locations	
Liver Brain Bone	5 (15.2) 10 (30.3) 17 (51.5)
Response to aumolertinib (N = 23)	
CR PR SD PD ORR DCR	0 (0) 15 (69.6) 6 (26.1) 2 (8.7) (65.2) (91.3)

Abbreviations: ECOG PS, eastern cooperative oncology group performance status; EGFR, epidermal growth factor receptor; CR, complete response; PR, partial response; SD, stable disease; PD, progressive disease; ORR, objective response rate; DCR, disease control rate.

### 3.2 Efficacy evaluation and safety profile

Before February 2024, 23 patients could be evaluated for treatment efficacy. The median follow-up time was 264 days (interval: 36–491 days). ORR and DCR were 65.2% and 91.3%, respectively. There was no significant difference in ORR between the major subgroups (Supplementary Table 1). In detail, 15 patients (65.2%) achieved PR, six (26.1%) achieved SD, and two patients (8.7%) experienced PD (Table 1). Due to the short follow-up period, median OS and PFS have not yet been reached (Supplementary Figure 1). The rate of treatment-related adverse events (TRAEs) and grade 3 or larger TRAEs was 87.9% and 12.1%, respectively. Detailed information is summarized in Supplementary Table 2.

### 3.3 PROs

**FIGURE 2:**
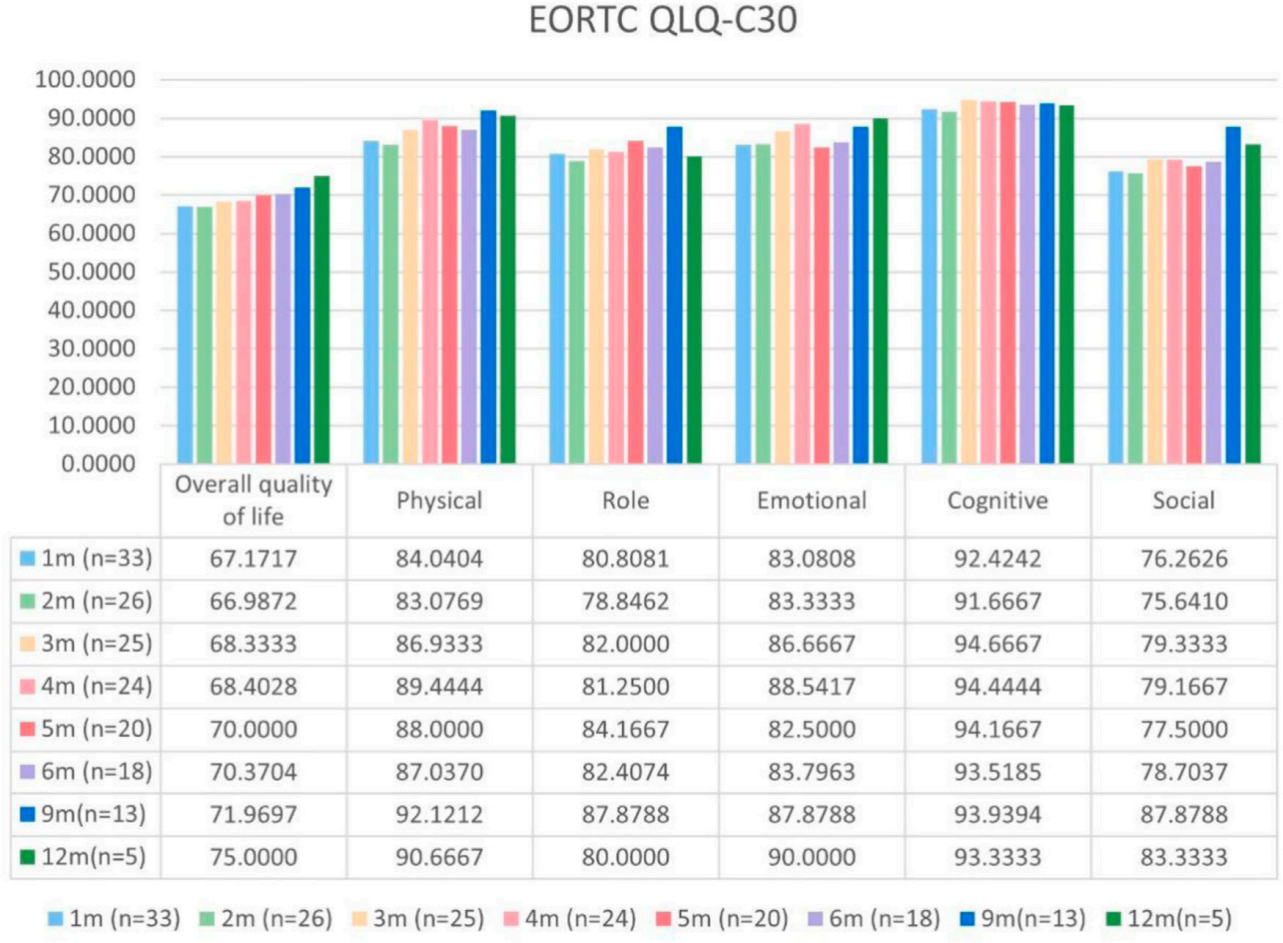
Mean scores of the general health status scale and functional scales from the European Organization for Research and Treatment of Cancer (EORTC) Quality of Life Questionnaire C30 (QLQ-C30) with various time points.

**FIGURE 3:**
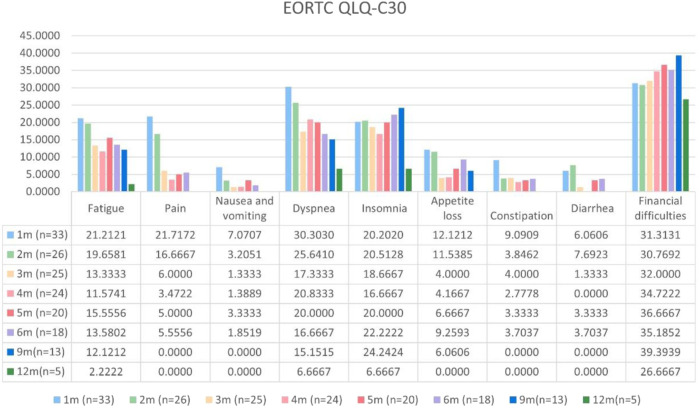
Mean scores of the symptom scales and items from the European Organization for Research and Treatment of Cancer (EORTC) Quality of Life Questionnaire C30 (QLQ-C30) with various time points.

**FIGURE 4:**
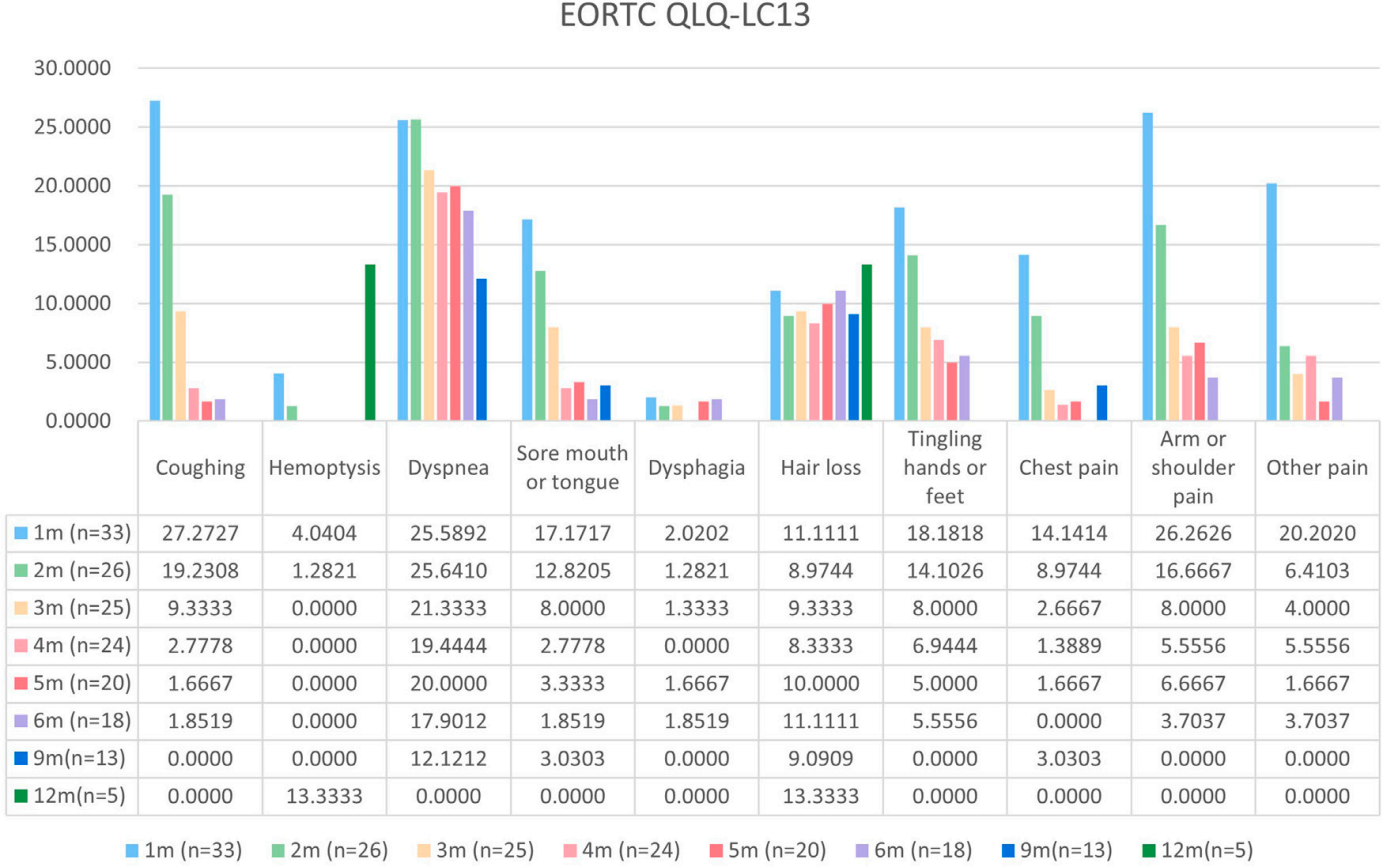
Mean scores of the symptom scales and items from the European Organization for Research and Treatment of Cancer (EORTC) Quality of Life lung cancer-specific module QLQ-LC13 with various time points.

**FIGURE 5:**
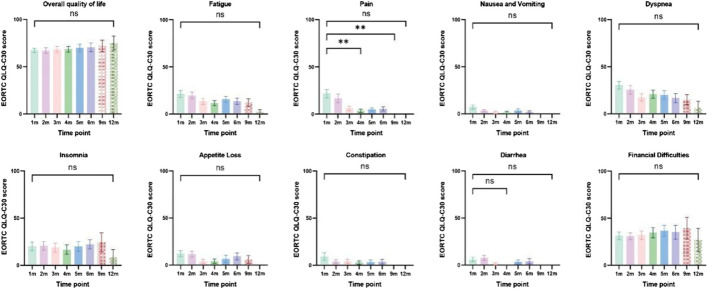
Column charts of general health status, three symptom scales, and multiple individual items. Statistical information: ns: non-significant, *p* > 0.05; "**", *p* < 0.01.

**FIGURE 6:**
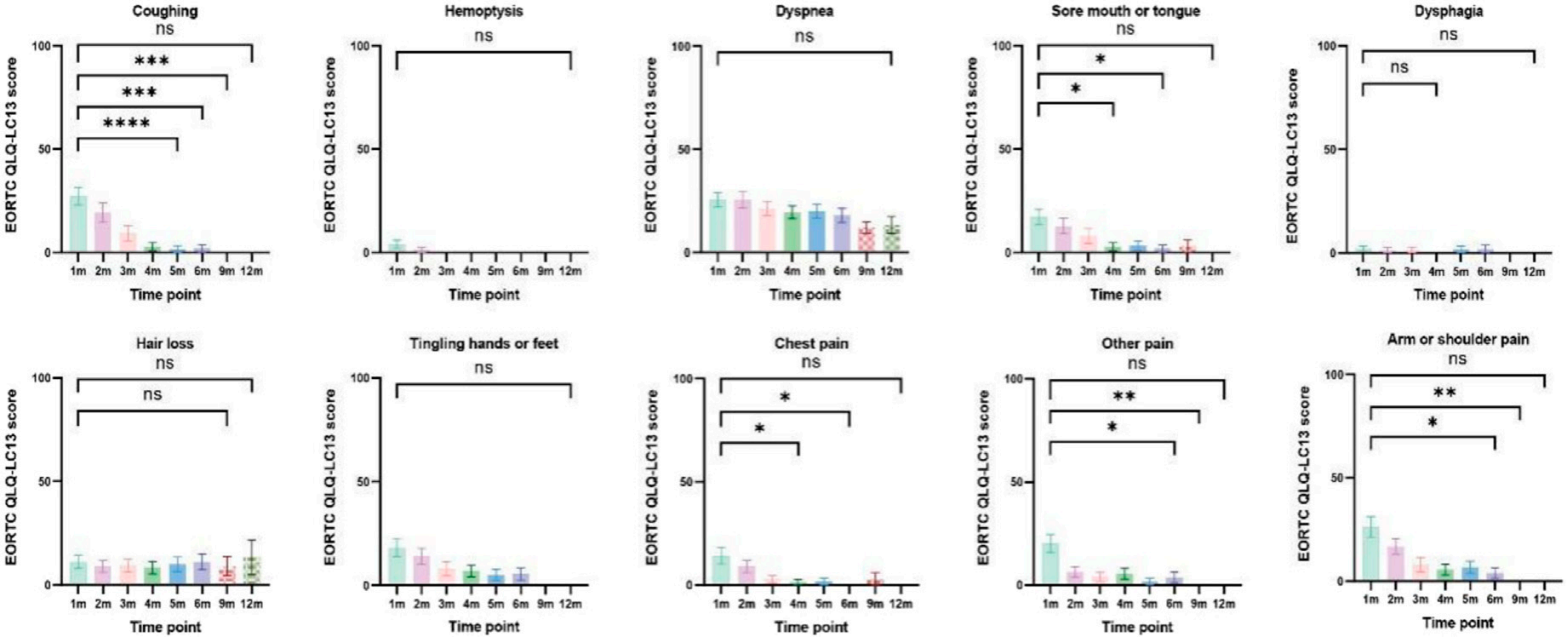
Column charts of lung-cancer-related symptoms and treatment-related adverse effects. Statistical information: ns: non-significant, *p* > 0.05; "*", *p* < 0.05; "**", *p* < 0.01; "***", *p* < 0.001; "****", and *p* < 0.0001.

Patients with at least one quality-of-life questionnaire were included in this analysis, and 54.5% of patients completed the first half-year follow-up. Patients reported a tendency toward higher functional scale scores, indicating good physical, role, emotional, cognitive, and social functioning after aumolertinib treatment (Figure 2). The overall quality of life score increased from 67.17 at baseline to 70.37 at the 6-month follow-up and 75.0 at the 12-month follow-up (Figure 2). The mean scores of the symptom scales and items also showed decreasing trends (Figure 3), indicating that aumolertinib treatment controlled symptoms. The mean scores of the first-month symptom scores in the aumolertinib arm were 21.21 for fatigue, 21.72 for pain, 7.07 for nausea and vomiting, 30.3 for dyspnea, 20.2 for insomnia, 12.12 for appetite loss, 9.09 for constipation, 6.06 for diarrhea, and 31.31 for financial difficulties (Figure 3). Compared to baseline scores, aumolertinib showed a clinically meaningful improvement in mean scores for pain and dyspnea after 6 months of treatment (Figure 3). However, only a decrease in pain scores was statistically significantly meaningful (Figure 4). QLQ-LC13 showed that lung-cancer-related symptoms also improved a lot, of which coughing, sore mouth or tongue, tingling in the hands or feet, chest pain, arm or shoulder pain, and other pain improvements were clinically meaningful at 6 months (Figure 5). Coughing, sore mouth or tongue, chest pain, arm or shoulder pain, and other pain improvements were statistically significantly meaningful (Figure 6).

## 4 Discussion

In the AENEAS trial, first-line treatment with aumolertinib demonstrated superior efficacy to gefitinib in advanced NSCLC patients with an activating EGFR mutation (Lu et al., 2022b). However, PROs have not been reported until now. In this real-world study, we evaluated the efficacy of aumolertinib with a particular focus on PROs. Our findings were consistent with those of the results reported in the AENEAS trial; importantly, we observed improvements in key lung cancer symptoms from baseline. Improvements in symptoms such as cough, sore mouth or tongue, tingling in the hands or feet, chest pain, arm or shoulder pain, and other types of pain were both clinically and statistically significant.

In the management of advanced NSCLC patients, incremental gains in PFS or OS are thought of as clinically meaningful only if they are achieved without a marked negative effect on HRQoL (Yang et al., 2015). Therefore, it is of great significance to record PROs in trials and real-world studies. The AENEAS trial showed a significant improvement in PFS of aumolertinib compared with gefitinib (19.3 months vs. 9.9 months) and a similar ORR of aumolertinib compared with gefitinib (73.8% vs. 72.1%) (Lu et al., 2022b). In this real-world study, the ORR of aumolertinib was 65.2%, which is comparable to that reported in clinical trials. The median PFS and OS were not reached because of a relatively shorter follow-up, and we will continue to track them. The similar short-term efficacy and demographics in our study support the following PRO analysis and may also reflect the results in AENEAS.

The EORTC QLQ-LC13 and QLQ-C30 questionnaires are well-established and are widely used in advanced NSCLC treatment trials (Geater et al., 2015; Bezjak et al., 2006; Blackhall et al., 2014; Brahmer et al., 2017; Yang et al., 2013), and have been thoroughly validated (Bergman et al., 1994; Aaronson et al., 1993; Sprangers et al., 1996). In this prospective and real-world study, questionnaire completion rates were high, with 54.5% of patients completing it during the first half year of aumolertinib treatment. Patients receiving first-line EGFR-TKI treatment usually have good performance status and low symptom burden, resulting in low symptom scores at baseline and difficulty in improvement measures. In practice, a score change equal to or greater than 10 points on the EORTC QLQ-LC13 and QLQ-C30 questionnaires is commonly deemed clinically significant (Fiteni et al., 2016). However, it has been shown that a lower, 5-point cut-off could also be clinically relevant. When the 5-point cut-off was administered here, fatigue, nausea and vomiting, dyspnea, appetite loss, and constipation score improvement at 6 months were clinically relevant.

There were several limitations in this real-world study. Firstly, it was a single-center study and the sample size was relatively small. With the development of EGFR-TKIs, advanced NSCLC patients with sensitive EGFR mutations have many choices, including gefitinib, erlotinib, afatinib, dacomitinib, osimertinib, furmonertinib, and befotertinib, which restricts the number of people receiving a specific drug. Secondly, the mPFS and mOS were not reached, and these patients are still in follow-up.

In conclusion, the PRO results from this real-world study showed improvements from baseline in key lung cancer symptoms in advanced NSCLC patients receiving aumolertinib as first-line therapy. Further follow-up of survival and symptom scores is ongoing.

## Data Availability

The original contributions presented in the study are included in the article/Supplementary Material; further inquiries can be directed to the corresponding authors.

## Supplementary material

SUPPLEMENTARY FIGURE S1 Kaplan–Meier survival curves of **(A)** overall survival in all evaluated patients and **(B)** progression-free survival in all evaluated patients.

SUPPLEMENTARY FIGURE S2 Subgroup analysis of significantly changed factors in EORTC QLQ-C30 and EORTC QLQ-LC13.

SUPPLEMENTARY TABLE S1 Subgroup analysis of ORR.

SUPPLEMENTARY TABLE S2 Summary of safety profiles in all patients receiving aumolertinib.
