# Supplementary material for: Efficacy and patient-reported outcomes in advanced non-small cell lung cancer patients receiving aumolertinib as first-line therapy: a real-world study
Source: Front Pharmacol. 2024 Sep 6;15:1444707. doi: 10.3389/fphar.2024.1444707 (PMC11422657; doi:10.3389/fphar.2024.1444707)

**Supplementary Table 1**. Subgroup analysis of ORR.

|  | PR | SD | PD | ORR(%) | P |
| --- | --- | --- | --- | --- | --- |
| Overall(n=23) | 15 | 6 | 2 | 65.2 | - |
| Age(n=23)  ＜65  ≥65 | 8  7 | 4  2 | 1  1 | 61.5  70.0 | 1.000 |
| Sex(n=23)  Female  Male | 11  4 | 4  2 | 2  - | 64.7  66.7 | 1.000 |
| Genetic mutation(n=20)  EGFR L858R  EGFR 19Del | 6  6 | 4  2 | 1  1 | 54.5  66.7 | 0.465 |

**Supplementary Table 2**. Summary of safety profiles in all patients receiving aumolertinib.

| Adverse events N (%) | All (N=33) |
| --- | --- |
| All TRAEs | 29(87.9) |
| Fatigue | 5(15.2) |
| Insomnia | 2(6.1) |
| Headache | 1(3.0) |
| Pruritus | 5(15.2) |
| Rash | 4(12.1) |
| Mouth ulcer | 4(12.1) |
| Appetite loss | 3(9.1) |
| Nausea and vomiting | 3(9.1) |
| Diarrhea | 9(27.3) |
| CK elevation | 6(18.2) |
| ALT elevation | 3(9.1) |
| AST elevation | 5(15.2) |
| GGT elevation | 4(12.1) |
| Hyperbilirubinemia | 5(15.2) |
| Hypoalbuminemia | 2(6.1) |
| ALP elevation | 2(6.1) |
| GDH elevation | 4(12.1) |
| Neutropenia | 4(12.1) |
| Anemia | 4(12.1) |
| Sore throat | 1(3.0) |
| Dyspnea | 4(12.1) |
| ≥G3 TRAEs  AST elevation  GGT elevation  Rash  Fatigue | 4(12.1)  1(3.0)  1(3.0)  1(3.0)  1(3.0) |

Abbreviations: TRAEs: treatment-related adverse events; CK: creatine phosphokinase; ALT: alanine aminotransferase; AST: aspartate aminotransferase; GGT: Gamma-Glutamyl Transferase; ALP: alkaline phosphatase; GDH: glutamate dehydrogenase; G3: Grade 3.

**Supplementary Figure 1.** Kaplan–Meier survival curves of (A) overall survival in all evaluated patients and (B) progression-free survival in all evaluated patients.


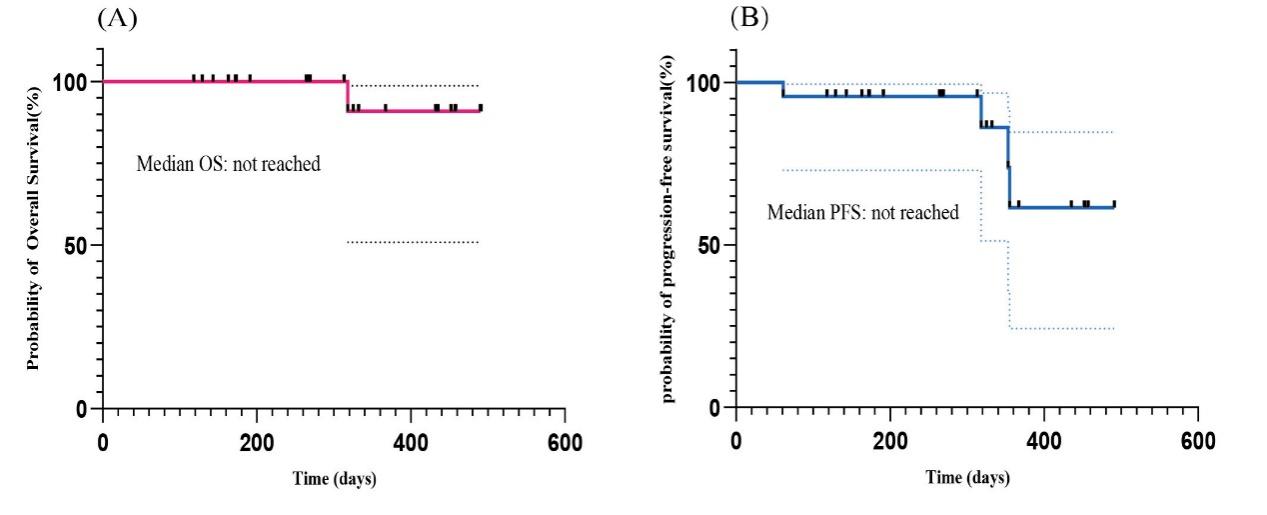


**Supplementary Figure 2**. Subgroup analysis of significantly changed factors in EORTC QLQ-C30 and EORTC QLQ-LC13.


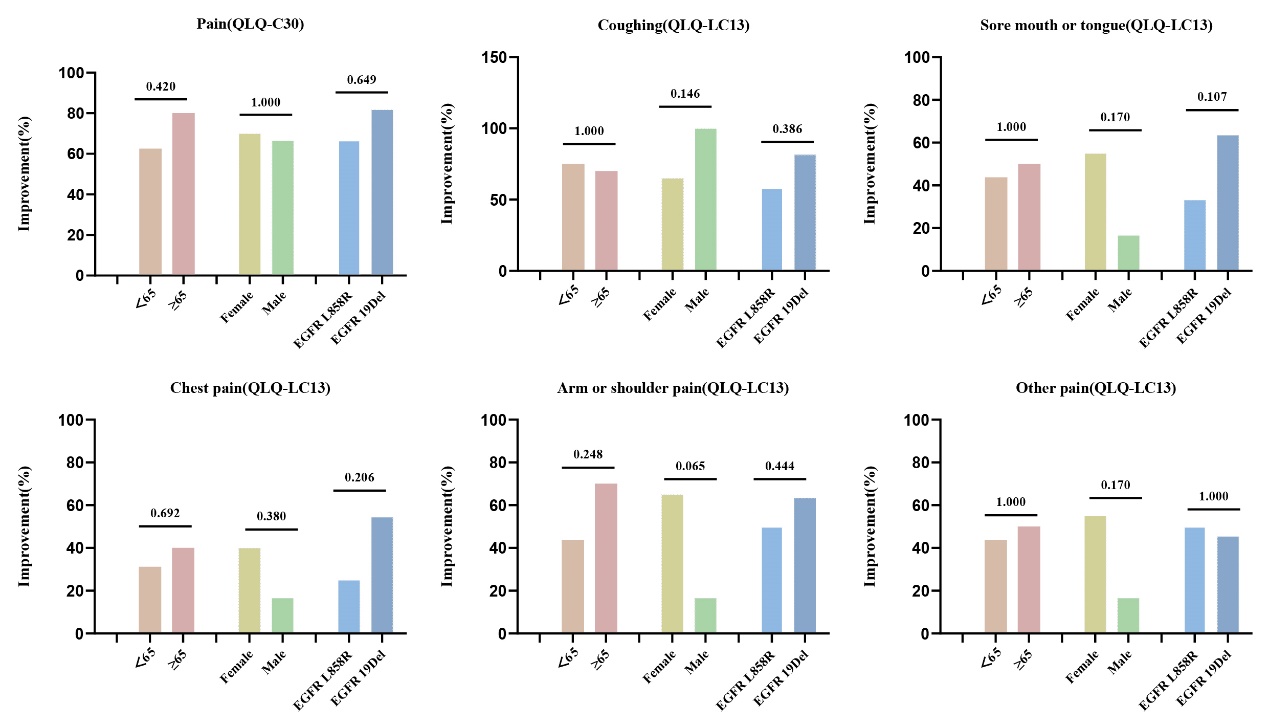

Supplement: Supplementary file 1 [file Table1.docx]
